# Gold Nanoparticles in Central Nervous System Diseases: Recent Progress, Challenges, and Therapeutic Opportunities

**DOI:** 10.1021/acs.nanolett.6c00540

**Published:** 2026-05-27

**Authors:** Shunping Han, Salima Bouchrara, Khuloud T. Al-Jamal

**Affiliations:** † Institute of Pharmaceutical Science, Faculty of Life Sciences & Medicine, 405987 King’s College London, Franklin-Wilkins Building, 150 Stamford Street, London SE1 9NH, United Kingdom; ‡ Department of Pharmacology and Pharmacy, Li Ka Shing Faculty of Medicine, The University of Hong Kong, Pokfulam 999077, Hong Kong, China

**Keywords:** Gold nanoparticles, Central nervous system diseases, Brain delivery, Therapeutic application, Toxicity

## Abstract

Central nervous system (CNS) diseases, including neurodegenerative disorders and brain cancers, remain leading causes of disability and death, largely due to limited drug penetration across the blood–brain barrier (BBB). Gold nanoparticles (AuNPs) have emerged as versatile platforms for CNS applications, with tunable morphology, surface chemistry, and optical properties, enabling both therapeutic and diagnostic use. This review highlights recent advances in the design and optimization of AuNPs for brain-targeted delivery, explores their applications in CNS diseases, and discusses current challenges, safety considerations, and future prospects for clinical translation.

## Graphical abstract

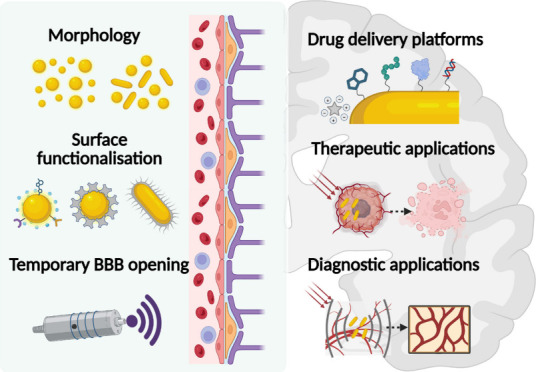

Central nervous system (CNS) diseases, including neurodegenerative disorders, cerebrovascular diseases, and brain cancers, remain major contributors to global disability and impose an escalating medical and socioeconomic burden. Brain cancers such as glioblastoma multiforme (GBM) remain highly aggressive and difficult to treat. Despite maximal surgical resection followed by Temozolomide-based chemoradiotherapy, the median survival for GBM remains less than 15 months, largely due to insufficient drug penetration across the blood–brain barrier (BBB) and high recurrence rates.^1^ In parallel, the prevalence of neurodegenerative diseases such as Alzheimer’s disease (AD) and Parkinson’s disease (PD) continues to increase, with many patients ultimately developing dementia and incurring substantial long-term societal and economic costs.^2^ Together, these challenges underscore a critical unmet need for innovative therapies and effective brain-targeted drug delivery systems.

Nanotechnology has emerged as a powerful approach to improve CNS drug delivery.^3^ A wide range of nanocarriers, including lipid-based, polymeric, and inorganic nanoparticles, have been investigated, with gold nanoparticles (AuNPs) receiving particular attention due to their unique physicochemical and optical properties. Depending on their morphology, AuNPs can take the form of gold nanospheres (AuNSs), nanorods (AuNRs), nanocages (AuNCs), nanoshells (AuNShs), nanostars (AuNSts), and other anisotropic AuNPs, with shape strongly influencing their physicochemical behavior and biological performance. Over the past two decades, AuNPs have been extensively explored in CNS disease applications, not only as delivery carriers but also as therapeutic and diagnostic agents.

This review provides an overview of recent advances in the design and optimization of AuNPs for brain delivery and explores their therapeutic potential in CNS diseases, with a particular emphasis on applications in neurodegeneration and brain tumors. In addition, it examines current limitations and safety concerns associated with AuNP-based interventions and outlines future perspectives to facilitate their clinical translation.

## Brain Delivery Efficiency: Quantitative Evaluation and Strategies for Enhancement

The efficiency of AuNP delivery to the brain is strongly influenced by the underlying health or disease state of the CNS, as pathological conditions may alter the permeability of the BBB. In addition, the administration route and intrinsic physicochemical properties of AuNPs, including size, shape, and surface functionalization, further modulate their accumulation in the brain (Figure 1).

**Figure 1:**
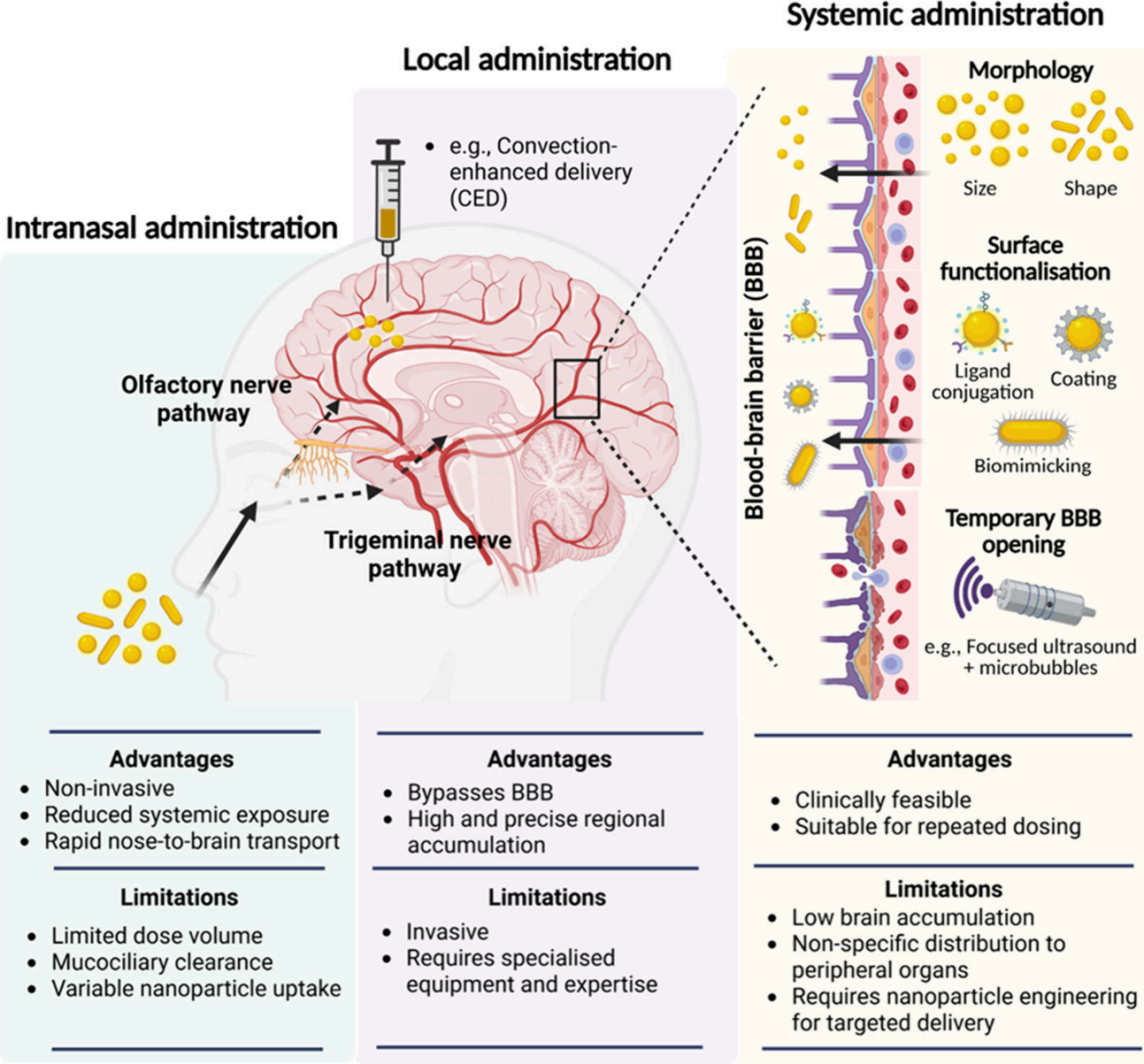
Strategies to enhance brain accumulation of AuNPs. Schematic illustration of the major administration routes for AuNP delivery to the brain, highlighting their respective advantages and limitations. The efficiency of AuNP brain delivery is influenced by both the route of administration and intrinsic physicochemical properties, including the particle size and shape. In addition, surface functionalization and complementary approaches, such as physical enhancement techniques, further modulate and enhance AuNP brain accumulation.

### Administration Route Determines the Brain Accumulation Efficiency of AuNPs

AuNPs have been administered to the brain through multiple routes in preclinical studies, including direct local injection, systemic delivery, and intranasal administration. Each route presents distinct advantages and limitations, which critically influence the efficiency of brain accumulation, as summarized in Figure 1. Brain local delivery enables AuNPs to bypass the BBB by depositing them directly into brain tissue or cerebrospinal fluid, achieving higher brain concentrations while minimizing systemic exposure and off-target effects. For example, a single cisterna magna injection of AuNPs in mice produced widespread CNS parenchyma distribution, with detectable signals persisting for several weeks.^4^ Convection-enhanced delivery of [^177^Lu] Lu-labeled AuNP, combined with anti-PD1 checkpoint immunotherapy, further improved survival in immunocompetent C57BL/6J mice bearing GL261 glioma.^5^ Despite their benefits, local delivery approaches are highly invasive, restricted to particular disease contexts, and pose challenges for clinical translation.

Intranasal administration offers a noninvasive route for direct brain targeting, largely exploiting the olfactory and trigeminal nerve pathways.^6^ Quantitative analyses revealed that intranasal delivery of AuNSs resulted in brain gold levels approximately 55-fold higher than those achieved by intravenous administration, without significantly altering regional brain distribution.^7^ Another study reported similar brain uptake of gold nanoclusters via intranasal and intravenous administration. Remarkably, intranasal delivery led to substantial accumulation in the nose, stomach, and intestines, whereas intravenous administration produced more than 10-fold higher uptake in major systemic organs, including the blood, lungs, liver, spleen, kidneys, and heart.^8^ Using multimodal imaging, our group further demonstrated that intranasally administered AuNRs entered the brain within minutes and progressively distributed to additional regions over 1 h, while maintaining minimal systemic exposure in healthy mice.^9^ When conjugated with neuroprotective peptides, these nanorods additionally exhibited neuroprotective potential.^10^

Despite encouraging preclinical outcomes with local and intranasal delivery, neither approach has yet reached clinical translation. At present, systemic administration, including intravenous and oral routes, remains the predominant strategy in clinical investigations of AuNP-based CNS therapies. However, systemic delivery faces a fundamental obstacle in the BBB, which restricts the entry of most therapeutics into the CNS and increases the risk of off-target toxicity. Strategies to optimize AuNP design for effective brain delivery will be discussed in the following sections.

### Morphology Influences Brain Delivery

In systemically administered AuNPs, particle size is a primary determinant of cellular uptake, circulation kinetics, organ tropism, and the ability to traverse biological barriers such as the BBB. Comparative studies of AuNPs ranging from 10 to 250 nm have shown clear size-dependent biodistribution patterns in healthy rodent models. The smallest particles exhibited the broadest tissue distribution following intravenous administration, with ultrasmall AuNPs (∼10 nm) detectable across all major organs. Importantly, these particles can cross the BBB, with approximately 0.3% of the injected dose (ID) detected in the brain 24 h postinjection by inductively coupled plasma mass spectrometry (ICP-MS). In contrast, larger AuNPs (>50 nm) exhibit limited systemic dispersion and are predominantly sequestered within the liver, spleen, and circulating blood, showing minimal to negligible brain accumulation.^11,12^ Structurally, the BBB is characterized by an extensive coverage of brain capillaries by astrocytic end-feet, separated from the endothelial surface by an intercellular gap of approximately 20 nm.^13^ This nanoscale structural gap offers a plausible mechanistic rationale for the enhanced ability of smaller AuNPs to cross the BBB.

Particle shape further modulates AuNP brain accumulation, although current evidence remains far more limited than that available for particle size. Studies using three-dimensional neurospheroid BBB models suggest that spherical AuNPs show restricted translocation across the endothelial barrier but penetrate more deeply once inside, whereas AuNRs cross the barrier more efficiently yet remain largely peripheral.^14^ Additional comparisons reveal that AuNSs and AuNRs of comparable size exhibited different interactions with plasma proteins and variations in macrophage uptake,^15^ potentially leading to variations in *in vivo* clearance and circulation time. In mice, PEGylated AuNSs (∼24 nm) and AuNRs (∼12 × 40 nm) with similar surface functionalization showed comparable brain accumulation (∼3.3% ID vs ∼2.5% ID, respectively) after systemic administration as quantified by inductively coupled plasma optical emission spectrometry (ICP-OES), while uptake in other major organs, such as the kidneys, spleen, liver, and lungs, was higher for nanospheres than for nanorods. Notably, longer nanorods (∼23 × 79 nm) showed diminished brain accumulation (0.5% ID) accompanied by increased liver and spleen uptake compared with shorter ones.^16^ These findings suggest that appropriately sized nanorods may serve as more efficient carriers for brain-targeted drug delivery, as they exhibit reduced accumulation in peripheral organs without compromising brain uptake. With respect to intranasal administration, an alternative route for brain delivery that may circumvent the BBB, particle morphology also appears to influence the efficiency of transport from the nasal cavity to the brain, although the available evidence remains limited. A direct comparison of gold nanoprisms and nanospheres with similar surface areas and modifications revealed that nanospheres achieved higher accumulation in the olfactory bulbs, greater systemic absorption, and substantially higher overall brain concentrations (∼3.01 ng Au/g tissue vs ∼0.54 ng Au/g tissue for nanoprisms).^7^ Despite these emerging insights, the scarcity of studies, coupled with differences in nanoparticle synthesis methods, animal models, and analytical techniques, likely contributes to inconsistencies across reported findings. A more comprehensive understanding of size and shape-dependent effects will be crucial for the rational design of AuNP-based platforms tailored for brain-targeted therapies.

### Surface Functionalization Enhances Brain Delivery

Surface functionalization provides an additional level of control, modulating AuNP biodistribution, enhancing targeting specificity, and ultimately governing their ability to reach brain tissue. Passive surface modifications primarily aim to prolong systemic circulation and increase the probability of AuNP interactions with the BBB. For instance, PEGylation reduces opsonisation and clearance by the mononuclear phagocyte system, consequently extending blood residence time and modestly enhancing brain accumulation. Similarly, polysorbate-80-stabilized AuNPs exhibited substantial brain uptake in mice 3 h after intravenous injection, with significant gold detected not only for 20 nm but also for 50 and 100 nm particles by ICP-OES, likely due to transient BBB modulation by polysorbate-80, including tight junction opening and temporary barrier disruption.^17^ Other biocompatible coatings, such as polydopamine, which provides strong adhesive interfacing, and glycol chitosan, which contributes a mild positive surface charge, have also been employed to facilitate intravenous delivery of AuNP to the brain.^18,19^

To achieve more precise and efficient brain delivery, conjugating targeting ligands to AuNPs has emerged as one of the most extensively explored strategies. By engaging receptor-mediated or adsorptive-mediated transcytosis pathways, ligand-functionalized AuNPs can actively exploit specific BBB transport mechanisms, thereby enhancing brain penetration and cellular specificity. In receptor-mediated transcytosis, ligands bind to specific receptors on brain endothelial cells, triggering clathrin- or caveolae-mediated endocytosis, followed by vesicular trafficking across the endothelial cytoplasm and subsequent exocytosis at the abluminal side. Receptors highly expressed on brain endothelial cells, including the transferrin receptor (TfR), low-density lipoprotein receptor (LDLR), and insulin receptor, have been widely investigated for nanoparticle-mediated brain delivery. Accordingly, AuNPs surface-functionalized with proteins (e.g., transferrin and insulin), peptides (e.g., angiopep-2), or antibodies (e.g., the 8D3 antibody) targeting these receptors have demonstrated enhanced brain accumulation in preclinical studies. For example, AuNRs conjugated with approximately 471 angiopep-2 peptides (ligand-targeting LDLRs) per particle exhibited a 7-fold increase in brain accumulation compared with PEGylated AuNRs (0.07% ID vs 0.01% ID), as quantitatively confirmed by neutron activation analysis.^20^ In parallel, adsorptive-mediated transcytosis is primarily driven by electrostatic interactions between positively charged ligands and the negatively charged luminal membrane, leading to nonspecific uptake and transcellular transport. Functionalization with cell-penetrating peptides, such as the transactivator of transcription (TAT) peptide, which primarily exploits adsorptive-mediated transcytosis, has been shown to enhance BBB permeability and promote efficient accumulation of AuNPs in brain tumors following systemic administration.^21^ Representative case studies of different targeting ligand functionalization are summarized in Table 1, illustrating that surface modification can substantially enhance brain accumulation of AuNPs, in some cases by several-fold. However, as observed in studies examining the influence of particle morphology on brain delivery, direct comparison of absolute brain concentrations across studies remains challenging due to variability and inconsistent or incomplete reporting of key physicochemical parameters, such as surface charge and targeting ligand density, as well as differences in experimental conditions, including animal models, dosing regimens, and quantification methods. To improve comparability, more standardized use of quantitative metrics and clearer reporting of experimental parameters would be beneficial.

**Table 1:** Surface Functionalization of AuNPs to Enhance Brain Delivery^a^

AuNP type	targeting ligand	size (nm)	ζ potential (mV)	func. efficiency (copies/AuNPs)	admin. route	quantification method	brain concentration/time point
AuNSs^22^	THR	∼37	–41	∼125	ip	INAA	∼0.07, 0.03, 0.02% ID at 1, 4, 24 h
AuNRs^20^	angiopep-2	∼118	–11	∼471	iv	NAA	∼0.07% ID (Angiopep-2-AuNRs) vs ∼0.01% ID (AuNRs) at 2 h
AuNSs^23^	insulin	∼20			iv	FAAS	∼5% ID (Insulin-AuNPs) vs ∼ 0.5% ID (AuNPs) at 2 h
AuNSs^24^	TfR-targeting antibody	∼77	–24	∼8	iv	ICP-MS	1.1% ID/g brain capillary vs 0.23% ID/g brain parenchymal at 8 h
AuNSs^25^	Enk neuropeptide	∼2		∼13	iv	γ counting	∼0.02% ID/g brain parenchymal (Enk-AuNPs) vs ∼0.007% ID/g brain parenchymal (AuNPs) at 4 h
						ICP-MS	0.07 ppm (Enk-AuNPs) vs <0.05 ppm (AuNPs) at 4 h
AuNSs^21^	TAT	∼21			iv	ICP-MS	∼2.9% ID (TAT-AuNPs) vs ∼0.6% ID (AuNPs) at 24 h

a Abbreviations: AuNRs, gold nanorods; AuNSs, gold nanospheres; FAAS, flame atomic absorption spectroscopy; ICP-MS, inductively coupled plasma mass spectrometry; INAA, instrumental neutron activation analysis; ip, intraperitoneal injection; iv, intravenous injection.

Extending beyond conventional passive and active targeting strategies, biomimetic surface engineering, particularly viral mimetics and cell-derived coatings, has emerged as a powerful approach to emulate physiological interfaces, thereby improving biocompatibility, stabilizing circulation, and facilitating BBB translocation. A representative example is the rabies virus (RABV), a prototypical neurotropic virus that gains CNS access through neuronal pathways and bypasses the BBB via its RABV glycoprotein. Inspired by this mechanism, RABV–mimetic, silica-coated AuNRs were developed and demonstrated markedly improved CNS distribution *in vivo*.^26^ Additionally, AuNPs cloaked with GBM patient-derived tumor cell membranes or neuron-targeted exosomes have shown strong BBB-crossing capabilities, further highlighting the promise of biomimetic coatings for CNS-directed delivery.^27,28^

### Other Strategies to Enhance Brain Delivery

Despite advances in morphological engineering and surface functionalization, systemically administered AuNPs still predominantly accumulate in peripheral organs, including the liver, lungs, and spleen. Consequently, additional strategies are being actively investigated to further enhance brain delivery.

Physical enhancement methods have been investigated as adjunct approaches to improve nanoparticle transport across the BBB. Magnetic resonance imaging guided focused ultrasound (MRgFUS) enables selective, focal, and transient BBB disruption. In a rat model, following intravenous administration, MRgFUS treatment of the right hemisphere resulted in more than a 3-fold increase in the delivery of 50 nm PEGylated AuNPs compared with the untreated left hemisphere, with silver-enhanced histology confirming nanoparticle localization within the brain parenchyma. Notably, after normalization to organ mass, AuNP accumulation in the treated hemisphere was comparable to that in the liver [∼1593 ng Au/g brain (right hemisphere) vs ∼1685 ng/g liver], indicating that MRgFUS substantially mitigates the typical liver–brain biodistribution imbalance.^29^ Similarly, in mice, focused ultrasound combined with microbubbles enhanced brain accumulation of intranasally administered AuNPs by 2.72-fold, with no evidence of hemorrhage in the treated brain.^8^ While these physical enhancement strategies have demonstrated substantial efficacy in enhancing AuNP transport across the BBB with an acceptable acute safety profile in preclinical models, their clinical translation will depend on rigorous optimization of safety parameters, improved predictability of delivery, and comprehensive long-term evaluation in human studies.

## Therapeutic Applications of AuNPs in CNS Diseases

Under appropriate design and surface functionalization, AuNPs have been shown to penetrate or bypass the BBB, accumulate in the brain, and selectively target specific pathological regions or cell types. In brain tumors, where the BBB is partially disrupted, nanoscale size-dependent enhanced permeability and retention (EPR) effects can further promote AuNP accumulation. Together with their unique optical properties and emerging intrinsic neuroprotective activity, these characteristics position AuNPs as a versatile platform for applications in CNS diseases, including drug delivery, photothermal therapy (PTT), diagnostic imaging, and neuroprotection (Figure 2).

**Figure 2:**
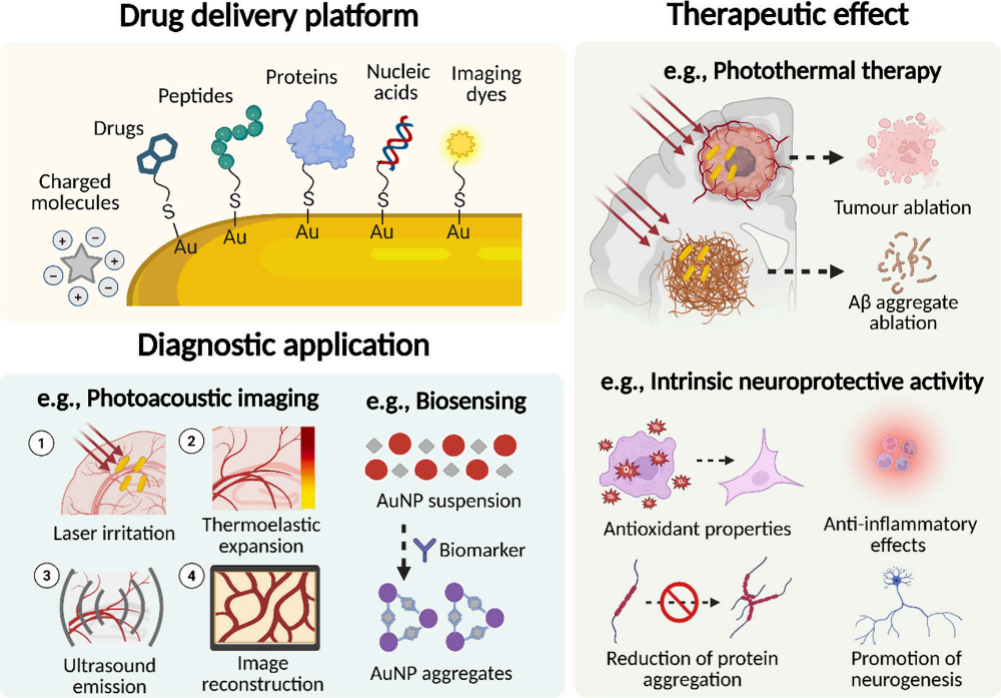
Therapeutic applications of AuNPs in CNS diseases. Due to their unique optical and surface properties, AuNPs have been extensively investigated as drug delivery platforms, therapeutic agents, and diagnostic modalities.

### Drug Delivery Carrier

The surface chemistry of AuNPs allows for the versatile conjugation of therapeutic agents through either noncovalent interactions, such as electrostatic adsorption, or covalent attachment mediated by functional groups such as thiols, amines, and carboxylates. Among these, thiol–gold (Au–S) bond exhibits the strongest binding affinity (∼40 kcal mol^–1^), exceeding that of Au–N (∼8 kcal mol^–1^) and Au–COO^–^ (∼2 kcal mol^–1^) interactions,^30^ making it the most widely employed strategy for the stable and efficient attachment of diverse therapeutic cargos.

As a powerful drug delivery platform, AuNPs have been employed to transport a wide range of therapeutic cargos, including chemotherapeutics [e.g., Temozolomide, doxorubicin (DOX), and cisplatin], therapeutic peptides (e.g., Acyl-ghrelin, CLPFFD peptides), and nucleic acids (e.g., Bcl2L12-specific small interfering RNA), demonstrating promising therapeutic potential in preclinical models of CNS disorders such as glioblastoma and neurodegenerative diseases. Chemotherapeutic agents are typically small hydrophobic molecules that primarily benefit from improved solubility and tumor accumulation upon AuNP conjugation. As a representative example, DOX was conjugated to AuNPs via an acid-responsive hydrazone linker and further functionalized with angiopep-2 to enhance brain and tumor targeting. Compared with free DOX, DOX–AuNP conjugates showed improved delivery efficiency, evidenced by enhanced glioma cell distribution after intravenous administration in glioma-bearing mice, primarily driven by the EPR effect and angiopep-2-mediated targeting. Notably, angiopep-2-modified PEGylated DOX-AuNPs (An-PEG-DOX-AuNPs) achieved higher tumor accumulation than PEG-DOX-AuNPs and free DOX, resulting in significantly prolonged median survival times (189%, 126%, and 47% increases, respectively, vs saline).^31^ In contrast, therapeutic peptides and nucleic acid cargos face more pronounced delivery challenges due to their larger size, negative charge, and susceptibility to proteolytic and nuclease degradation. Formation of nanocomplexes can substantially improve their stability by protecting them from enzymatic degradation and facilitating cellular uptake via endocytosis, thereby providing a complementary strategy to overcome key delivery barriers.^32^ A representative example is NU-0129, in which radially oriented, densely packed small interfering RNA (siRNA) oligonucleotides are covalently conjugated to a AuNP core, forming a spherical nucleic acid architecture that enhances nuclease resistance, enables efficient delivery to glioblastoma, and has progressed into early phase clinical trials.^33^ In PD models, AuNPs loaded with plasmid DNA designed to suppress α-synuclein expression and functionalized with docosahexaenoic acid (DHA) and nerve growth factor (NGF) to facilitate BBB penetration and neuronal targeting, significantly enhanced tyrosine hydroxylase (TH) expression while reducing α-synuclein aggregation in the substantia nigra. This treatment alleviated motor deficits, restored exploratory behavior, and reversed long-term potentiation (LTP) impairments, indicating improvement in both motor and nonmotor functions.^34^

While these studies demonstrate promising therapeutic outcomes, their efficacy is fundamentally governed by complex and not yet fully resolved transport processes across the BBB. In particular, the interplay between protein corona formation and cellular uptake mechanisms, including endocytic pathways and receptor-mediated transcytosis requires further systematic investigation using advanced experimental platforms, such as human *in vitro* BBB models, complemented by *in vivo* imaging approaches. Integrating such mechanistic insights with quantitative pharmacokinetic and pharmacodynamic correlations will be essential for establishing predictive design principles and advancing rational CNS-targeted AuNP therapeutics.

### Photothermal Therapy (PTT)

Tunable localized surface plasmon resonance (LSPR) is another central property that underlies the biomedical utility of AuNPs. LSPR originates from the collective oscillation of conduction electrons at the nanoparticle surface under electromagnetic excitation, resulting in strong and wavelength-selective optical absorption. By controlling the size and shape of AuNPs, the LSPR of AuNPs (e.g., AuNRs, AuNCs, and AuNShs) can be tuned into the biological transparency window (650–1350 nm), where light penetration in tissues is maximized. This tunability enables efficient photothermal conversion, positioning AuNPs as versatile and effective agents for PTT.

Photothermal strategies have been extensively applied in cancer therapy, where targeted heating can selectively ablate malignant cells. As mentioned in the previous section, leveraging biomimetic surface engineering, RABV–mimetic, silica-coated AuNRs achieved efficient accumulation in the brain. Upon 808 nm near-infrared (NIR) laser irradiation, these nanorods generated a localized hyperthermal effect, effectively suppressing tumor growth in both xenograft and orthotopic brain tumor mouse models.^26^

Beyond oncology, the photothermal properties of AuNPs are increasingly explored for neurodegenerative diseases, particularly AD. Localized heating can disrupt amyloid-β (Aβ) aggregates, key pathological hallmarks of AD, and mitigate neurotoxicity. For example, AuNRs functionalized with Aβ-targeting inhibitory peptides and capped with ceria nanoparticles combine precise targeting with antioxidant protection. Upon 808 nm NIR laser irradiation, these nanocomposites exhibit excellent photothermal stability and conversion efficiency. In APP/PS1 transgenic mice, they improved cognitive function by inhibiting Aβ monomer aggregation, promoting disassembly of Aβ fibrils, and reducing oxidative stress through ROS scavenging, underscoring the potential of PTT as a direct, multimodal intervention for AD.^35^

Despite promising preclinical results, AuNP-based PTT has not yet advanced to clinical studies for CNS diseases. A major translational barrier arises from the combined effects of optical attenuation in biological tissues and safety constraints on NIR irradiation. For effective PTT, NIR light must traverse the scalp, skull, and healthy brain tissue to reach target sites without causing damage. However, due to substantial anatomical differences, millimeter-scale penetration is typically sufficient in small animal models, whereas the centimeter-scale thickness of the human skull and brain leads to severe attenuation of NIR energy, preventing effective and localized heating. This limitation is further compounded by the high sensitivity of neural tissue to thermal injury. Clinically, NIR exposure is restricted by established safety standards, including those defined by the American National Standards Institute (ANSI) and the International Commission on Non-Ionizing Radiation Protection (ICNIRP), which specify wavelength- and time-dependent maximum permissible exposure (MPE) limits. In translational photothermal studies, power densities are typically maintained within the conservative range of 0.2–0.5 W/cm^2^ for continuous-wave irradiation to ensure safety and avoid thermal damage to superficial tissues. Consequently, increasing laser power to compensate for deep-tissue attenuation would exceed safety thresholds at the tissue surface, rendering such approaches clinically impractical. To address these challenges, emerging strategies focus on enhancing photothermal conversion efficiency, developing minimally invasive light delivery approaches (e.g., intracranial optical fiber implantation), and integrating PTT with complementary therapeutic modalities to reduce the required thermal dose and improve overall efficacy, thereby facilitating clinical translation.

### Diagnostic Applications

Due to the LSPR, AuNPs have also been extensively employed as photoacoustic (PA) imaging contrast agents. Upon pulsed laser irradiation, AuNPs efficiently absorb optical energy and rapidly convert it into heat, resulting in thermoelastic expansion and the generation of broadband acoustic waves that can be detected to reconstruct high-resolution images. Compared with fluorescence optical imaging, PA imaging offers improved spatial resolution and greater imaging depth, due to the substantially reduced scattering of ultrasonic waves relative to light in biological tissues. Depending on the imaging configuration, PA imaging can achieve micrometre-scale resolution or centimeter-scale penetration depth. In contrast to conventional ultrasound imaging, which derives contrast primarily from the mechanical properties of tissues, PA imaging provides contrast based on optical absorption, enabling enhanced differentiation of biological structures. Furthermore, the absence of ionizing radiation renders PA imaging inherently safer than computed tomography (CT) and radionuclide-based modalities such as positron emission tomography (PET) and single-photon emission computed tomography (SPECT).^36^ AuNP-based PA imaging has been applied to visualize cerebral vasculature and assess functional parameters in small-animal models.^36^ However, similar to PTT, the translation of AuNP-based PA imaging to CNS applications is constrained by light delivery, limiting imaging depth and signal-to-noise ratio under safe irradiation conditions and potentially necessitating higher nanoparticle doses. Moreover, potential structural and optical instabilities under repeated laser excitation, along with unresolved issues regarding biodistribution, clearance, and long-term neurotoxicity, further impede translation.^36^ Beyond PA imaging, AuNPs have also shown promise in other CNS imaging modalities, including computed tomography and surface-enhanced Raman scattering (SERS).

In addition to imaging, the distinctive optical and surface properties of AuNPs make them highly attractive for biosensing applications. AuNP-based sensors have been developed to detect a broad range of neurological disease-related biomarkers, including amyloid-β, tau protein, and acetylcholinesterase (AChE), using diverse signal transduction modalities such as colorimetric, electrochemical, fluorescence, light-scattering, and SERS-based readouts. A representative example is the use of rhodamine B-functionalized AuNPs (RB-AuNPs) as a dual-mode biosensor for quantitative AChE detection, a key biomarker of AD. In this system, RB-AuNPs are initially well dispersed. Upon the addition of acetylthiocholine (ATC), an analogue of acetylcholine, and AChE to the RB-AuNP solution, AChE catalyzes the hydrolysis of ATC to generate thiocholine, whose thiol groups exhibit strong affinity for the Au surface and displace RB via ligand exchange. This process induces AuNP aggregation, leading to a distinct color change from red to purple, while simultaneously restoring RB fluorescence as the dye is released from the AuNP surface, enabling dual colorimetric and fluorescent readouts.^37^

### Neuroprotection

Independent of their role as delivery or diagnostic platforms, AuNPs possess intrinsic biological activities with emerging therapeutic relevance for CNS diseases, as highlighted in recent reviews.^38,39^ Mechanistically, AuNPs can attenuate ROS-driven oxidative stress, a key contributor to neurodegeneration, partly through activation of antioxidant signaling pathways such as the Nrf2-ARE axis, thereby restoring intracellular redox homeostasis.^38^ In parallel, they exert anti-inflammatory effects by modulating key signaling cascades, including NF-κB and MAPK pathways, which can suppress microglial and astrocytic activation and reduce the production of pro-inflammatory cytokines such as interleukin-1β (IL-1β) and tumor necrosis factor-α (TNF-α).^39^ At the organelle level, AuNPs have been reported to stabilize mitochondrial membrane potential, improve mitochondrial respiration, and enhance ATP production, supporting cellular energy homeostasis and neuronal viability.^38,40^ These effects are closely associated with modulation of ROS homeostasis and downstream apoptotic signaling cascades. AuNPs can additionally interfere with the aggregation of misfolded proteins, including Aβ in AD and α-synuclein in PD, thereby preserving neuronal structure and function.^41,42^ Notably, emerging evidence suggests that electromagnetised AuNPs may promote neurogenesis and improve cognitive performance in the aged brain.^43^

Collectively, these multifaceted actions contribute to neuroprotection by attenuating chronic neuroinflammation, oxidative damage, and other pathological processes underlying neurodegenerative disorders. It is important to note that these mechanisms are highly context-dependent, varying with AuNP size, shape, surface chemistry, synthesis methods, and the specific neurological condition under investigation.

## Clinical Status

Although AuNPs have demonstrated substantial promise in preclinical models of neurological disorders, clinical translation remains limited. To date, evidence for AuNP-based platforms in CNS diseases is largely confined to a small number of early phase studies, most notably CNM-Au8 and NU-0129, with only sporadic additional exploratory applications reported. CNM-Au8 is a suspension of faceted, clean-surfaced gold nanocrystals engineered to enhance neuronal energy metabolism and support remyelination.^44^ Mechanistically, CNM-Au8 is proposed to catalytically oxidize NADH to NAD^+^, potentially via the high surface reactivity of its faceted gold nanocrystals, thereby increasing the intracellular NAD^+^/NADH ratio.^45^ This shift in redox balance is hypothesized to enhance mitochondrial bioenergetics, leading to increased ATP production and improved metabolic function in neurons and oligodendrocytes, which may contribute to remyelination. CNM-Au8 has been evaluated in several phase 2 studies across multiple sclerosis (MS), PD, and amyotrophic lateral sclerosis (ALS). In the REPAIR-MS (NCT03993171) and REPAIR-PD (NCT03815916) trials, oral administration of CNM-Au8 increased the brain NAD^+^/NADH ratio, indicating CNS-relevant bioenergetic modulation.^44^ In the Phase 2 VISIONARY-MS trial (NCT03536559 and NCT04626921) involving patients with stable relapsing-remitting MS and chronic optic neuropathy, CNM-Au8 demonstrated trends toward functional improvement, including changes in low-contrast visual acuity and modified MS functional composite (mMSFC) scores. Exploratory imaging and physiological assessments provided preliminary evidence suggestive of remyelination, and the therapy was generally well tolerated. In ALS, CNM-Au8 was evaluated in the RESCUE-ALS trial (NCT04098406), showing trends toward clinical benefit and consistent tolerability.

Another notable example is NU-0129, a spherical nucleic acid nanoconstruct comprising a gold core densely functionalized with radially oriented siRNA oligonucleotides.^33^ In a first-in-human trial (NCT03020017), NU-0129 was administered intravenously to patients with recurrent GBM or gliosarcoma. This study provided the first direct evidence that gold-based nanoparticles can traverse the BBB and accumulate within human brain tumors without inducing significant toxicity.

Overall, these studies collectively suggest the feasibility of CNS delivery, target engagement, and acceptable safety profiles in humans, thereby establishing an initial translational foundation for future AuNP-based therapeutics in neurodegenerative, demyelinating, and malignant CNS diseases.

## Safety Considerations and Future Perspectives of AuNPs in CNS Applications

Although AuNPs are generally considered biocompatible, safety concerns remain a major obstacle to their clinical translation for CNS therapies. Several studies have reported instances of CNS adverse effects. AuNPs have been shown to disrupt the integrity of tight junctions in mouse brain microvascular endothelial cells *in vitro* and to increase BBB permeability *in vivo*.^46^ Investigations into size-dependent neurotoxicity have revealed that stereotactic injection of 5 nm AuNPs into the cerebral cortex induced higher expression of nestin, a marker of CNS injury, compared with 100 nm AuNPs, indicating that smaller nanoparticles may exhibit greater toxicity.^47^ In addition to size, particle shape and surface chemistry also influence neurotoxic outcomes. Comparative studies of spherical, rod-shaped, and urchin-like AuNPs with identical surface coatings (PEG or CTAB) have shown that spherical AuNPs, particularly CTAB-coated ones, markedly reduce mitochondrial metabolic activity, whereas rods and urchins were less toxic at the tested concentrations. Following intranasal administration in transgenic mice, AuNPs induced transient microglial activation and upregulation of toll-like receptor 2 (TLR-2) in the olfactory bulb in a shape- and surface-dependent manner. Spherical AuNPs caused sustained activation, rod-shaped AuNPs elicited a biphasic response, PEGylated urchins showed no significant effect, and CTAB-coated urchins caused only transient activation.^48^ Furthermore, AuNP-induced neurotoxicity is highly dependent on dose and exposure duration. Rats that received a single intravenous injection of PEG-coated AuNPs (100 μg/kg body weight) did not induce measurable inflammatory, oxidative stress, or apoptotic responses over the observation period. In contrast, citrate-capped AuNPs produced no detectable acute toxicity at 2 weeks, but led to a significant increase in pro-inflammatory cytokines (TNF-α and IL-6) in brain tissue after 12 weeks, indicating delayed neuroinflammatory effects.^49^ Similarly, evidence from subchronic exposure shows that repeated intraperitoneal administration of 10 nm AuNPs (2 mg/kg/day for 28 days) resulted in widespread histological damage in the cortex, hippocampus, and cerebellum, including neuronal degeneration, vascular alterations, and glial activation. These changes were associated with decreased Beclin-1 expression (impaired autophagy) and increased GFAP and caspase-3 levels, indicating enhanced astrogliosis and apoptosis.^50^ Intraperitoneal administration of 20 nm AuNPs at 20 μg/kg for 3 days induced a significant decrease in the levels of neurotransmitters like dopamine and serotonin, indicating a possible change in the behavior of the treated animals.^51^ Notably, toxicity profile and clearance kinetics also showed species-dependent variations.^52^ Following a single intravenous injection (1000 mg/kg), mice developed hepatic granulomas and a transient increase in the pro-inflammatory cytokine interleukin-18, consistent with a macrophage-mediated response. In contrast, rats exhibited no comparable inflammatory or histopathological changes but showed acute sensitivity, with several animals dying within hours of administration. These divergent outcomes were accompanied by distinct biodistribution and excretion patterns, with rats displaying higher splenic accumulation and greater faecal elimination of AuNPs. Overall, the potential harmful effects of AuNPs in biological systems are influenced by factors such as particle size, shape, surface coating, dosage, administration approach, exposure regimen and species. Establishing a universal toxicity threshold for AuNPs remains challenging, underscoring the need for systematic studies incorporating standardized chronic exposure paradigms with integrated multilevel end points and clearance kinetics to comprehensively evaluate their biological impact and improve translational relevance.

Beyond biological considerations, the clinical translation of AuNP-based CNS nanomedicines is constrained by unresolved manufacturing and standardization issues. Compliance with Good Manufacturing Practice requires strict control of nanoparticle size, morphology, surface chemistry, ligand density, and endotoxin contamination, as even subtle variations can significantly alter pharmacokinetics and brain distribution. Moreover, insufficient characterization of aggregation and protein corona formation in biological media remains a major source of irreproducibility across studies. In particular, surface chemistry reproducibility is problematic, as variability in ligand density and stability directly affects BBB interactions and cellular uptake, contributing to inconsistent *in vivo* outcomes. In addition, sterilization procedures (e.g., autoclaving, filtration, and γ irradiation) may unintentionally modify nanoparticle structure and induce aggregation or ligand loss, further compromising performance. For photothermal systems, stability under laser irradiation should also be systematically validated. From a regulatory standpoint, robust assessment of biodistribution, CNS penetration, off-target accumulation, long-term retention, and immunogenicity is essential for clinical translation. Collectively, these limitations highlight the lack of standardized quality and safety frameworks, representing a critical barrier to clinical advancement of AuNP-based CNS therapies.
